# Preparation of RGO/TiO_2_/Ag Aerogel and Its Photodegradation Performance in Gas Phase Formaldehyde

**DOI:** 10.1038/s41598-019-52541-7

**Published:** 2019-11-08

**Authors:** Haiwang Wang, Guanqi Wang, Yukai Zhang, Yuan Ma, Zhengjie Wu, Dekuan Gao, Rutong Yang, Bingzhu Wang, Xiwei Qi, Jun Yang

**Affiliations:** 1 School of Resources and Materials, Northeastern University at Qinhuangdao, Qinhuangdao, 066004 PR China; 2 School of Materials Science and Engineering, Northeastern University, Shenyang, 110819 PR China; 3 Key Laboratory of Dielectric and Electrolyte Functional Material Hebei Province, Qinhuangdao, PR China; 4 Institute of Process Engineering, Chinese Academy of Sciences, No. 1 North Second Street, Zhongguancun, Beijing, 100190 China

**Keywords:** Photocatalysis, Photocatalysis, Nanoparticles, Nanoparticles

## Abstract

To increase the utilization ratio and catalytic efficiency of the nano TiO_2_, The RGO/TiO_2_/(Ag) powders and RGO/TiO_2_/Ag aerogel photocatalyst were designed and prepared. The composition and microstructure of RGO/TiO_2_/(Ag) powders and RGO/TiO_2_/Ag aerogel were studied, in addition, the photocatalytic activity of RGO/TiO_2_/(Ag) powders and RGO/TiO_2_/Ag aerogel was researched by the photocatalytic degradation behavior of formaldehyde solution and formaldehyde gas respectively. The result indicate that TiO_2_ is uniformly loaded on the surface of RGO with a particle size of 10 nm to 20 nm. When the amount of graphene oxide added is 1 wt%, RGO/TiO_2_ powder has the highest degradation effect on formaldehyde solution, in addition, the introduction of Ag can greatly improve the photocatalytic effect of the sample. The results also show that the pore size of RGO/TiO_2_/Ag aerogel is between 7.6 nm and 12.1 nm, and the degradation rate of formaldehyde gas is 77.08% within 2 hours.

## Introduction

With the great development of industry, formaldehyde residue in air and water is also increasing gradually^1,2^. Because of the wide presence of formaldehyde, which is still highly carcinogenic and sometimes teratogenic^3,4^, how to remove it efficiently becomes a significant issue. Compared with other treatment measures^5–7^, the formaldehyde degradation technology by using photocatalyst has become increasingly mature, and gradually attracted people’s attention^8–10^.

Due to its excellent photocatalytic performance and chemical stability, as well as relatively low price^11–13^, titanium dioxide is now the most widely used photocatalyst material^14–16^. However, in the conventional application process, as the photogenerated electron-hole pairs are prone to be recombined, the utilization of light energy is therefore not very efficent^17–19^. At the same time, the direct use of titanium dioxide powder as photocatalyst has the downside that the catalyst cannot be dispersed evenly and recovered easily. The above factors prevented titanium dioxide to be further applied in the industry.

In order to overcome the above disadvantages of TiO_2_, the noble metal (such as Ag) particles were loaded on the surface of titanium dioxide and the electron potential well was formed to promote the separation of electron hole pairs^20–22^. Meanwhile, the combination of titanium dioxide with carbon-based materials (such as RGO) with strong conductivity and electron capture performance can improve the transfer of photogenerated electrons, and further reduce the recombination and increase the photocatalytic reaction area^23–27^. Chao Chen *et al*. prepared a new type of TiO_2_/ chitosan /RGO system with highly distributed macromoledule structure by ice template method, and applied it to photocatalytic degradation. The results showed that the combination of TiO_2_ with RGO can promote the separation of electrons from the hole pairs in photochemical reaction and greatly improve the degradation rate of methyl orange^28^. Besides, in the previous work, we also used RGO/TiO_2_ powder to prepare RGO/TiO_2_-PAM composite flocculant via photocatalytic surface initiated polymerization method and found that RGO/TiO_2_ had great photodegradation performance in polyacrylamide^29^. Juan Li *et al*. introduced RGO into titanium dioxide and studied the influence of RGO on the charge transfer rate of titanium dioxide. The results showed that the TiO_2_/RGO hybrid agent loaded with RGO showed excellent charge transfer rate and excellent circulation ability^30^.

Although the above technologies are relatively mature, most of the existing research has focused only on the modification of titanium dioxide powder. And there are few researches on the improvement of the surface area of titanium dioxide and its composites and their recovery and reuse capabilities.

Aerogels have strong adsorption capability and strong construction, which can be used as the load framework of photocatalyst, and have a wide application prospect^30,31^. As far as we know, there is no corresponding report on the preparation of RGO/TiO_2_/Ag aerogels as photocatalyst. Therefore, the design and preparation of such heterogeneous structure in photocatalytic field will be of great significance^32^.

In this paper, a novel RGO/TiO_2_/Ag aerogels photocatalyst was prepared by freeze drying technology. By utilizing the loose and porous structure of aerogels, the specific surface area of the photocatalyst was increased while it was still easy to be recycled, further improving its application prospect in the degradation of gaseous formaldehyde.

## Results

### Flow chart of preparation of photocatalyst

**Figure 1:**
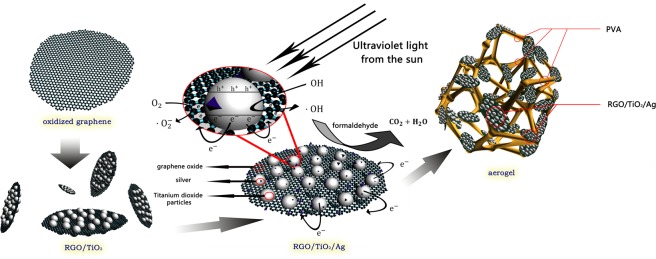
The producing process and operation mechanism of RGO/TiO_2_/Ag photocatalyst aerogel.

The design process of RGO/TiO_2_/Ag aerogel is shown in Fig. 1. The titanium dioxide dry gel is loaded onto the surface of graphene oxide by sol-gel method. By adjusting the heat treatment temperature in the crystallization process of titanium dioxide, TiO_2_ is loaded on the surface of RGO in the form of nanocrystals. With UV-lighting, Ag^+^ ions were reduced by photogenerated electrons generated on the surface of RGO/TiO_2_, so that silver nanoparticles deposited on the surface of RGO/TiO_2._ Finally, RGO/TiO_2_/Ag composite aerogel was prepared by freeze drying technology.

### Thermal analysis of titanium dioxide gel and graphene oxide composite

**Figure 2:**
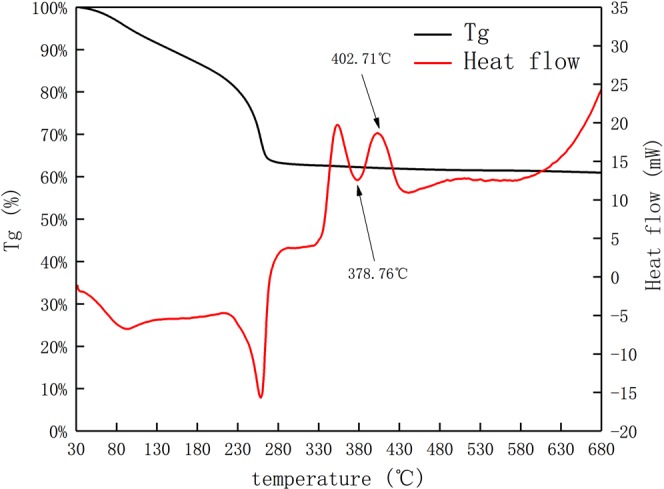
Thermogravimetric Analysis and Heat Flux Density Curve of RGO/TiO_2_ Composite gels.

Figure 2 shows the thermal analysis of the titanium dioxide gel and graphene oxide composite in nitrogen atmosphere. Among them, we can see that the sample loses weight at 30–180 °C, and forms a distinct endothermic peak in the heat flux curve. This is due to the evaporation of water and n-butanol produced during the hydrolysis of butyl titanate in the sample. The endotherm occurs at 230–280 °C, which may be due to decomposition of the incompletely hydrolyzed butyl titanate, and the sample quality will be reduced. An endothermic peak at 378.76 °C and an exothermic peak at 402.71 °C are also shown in Fig. 2, which represent the nucleation and crystallization temperatures of the anatase TiO_2_ grains respectively. As a result, the heat recovery temperature was set to 400 °C to promote nucleation, prevent the TiO_2_ particles from growing fastly, and make the nanocrystal structure uniform.

### XRD analysis of RGO/TiO_2_ and RGO/TiO_2_/Ag powers

**Figure 3:**
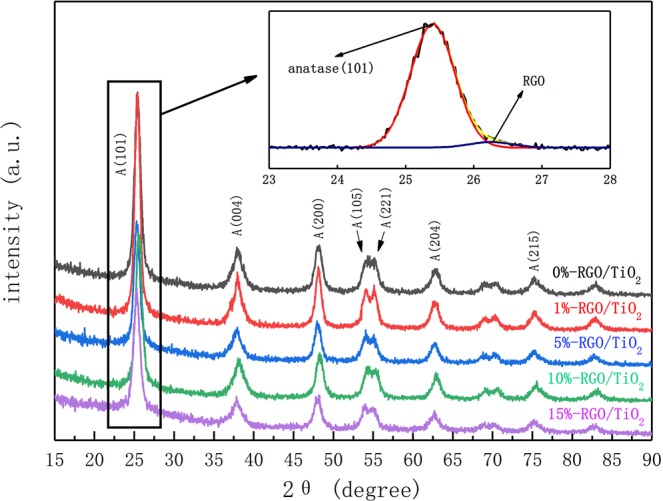
XRD comparison diagram for 3 types of RGO/TiO_2_ photocatalysts with crystalline faces remarks for major characteristic peaks, as well as the enlarged XRD diagram of peak split and combination for characteristic peaks where 2θ = 23–28.

Figure 3 is XRD patterns of RGO/TiO_2_ powders with different RGO content and it can be seen from the figure that the TiO_2_ shows obviously pointed characteristic peaks where 2θ = 25.42, 37.95, 48.11, 54.10, 55.13, 63.12 and 75.32, which correspond with the (101), (004), (200), (105), (221), (204) and (215) crystal faces of anatase TiO_2_. The crystalline grain radius of TiO_2_ can be calculated by using the Debye-Scherrer equation.

1 $${\rm{D}}=\frac{K\gamma }{B\,\cos \,\theta }$$

It can be known from the calculation of (1), the grain radius of TiO_2_ in photocatalyst was 11.067 nm, which means the TiO_2_ is loaded onto the graphene as nanocrystalline construction.

**Figure 4:**
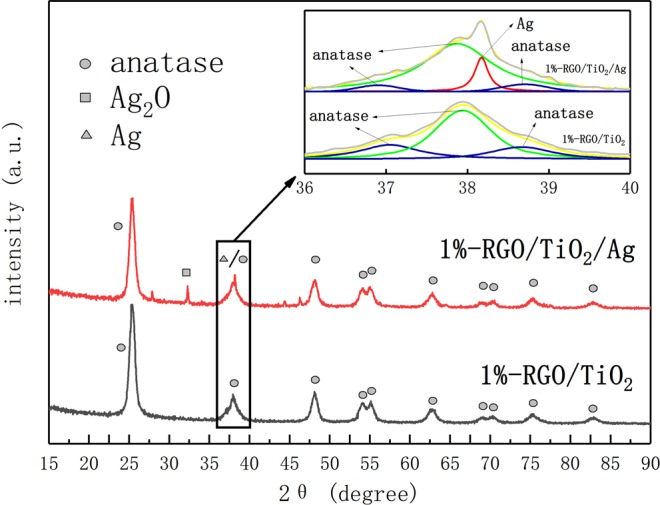
XRD Comparison diagram for 1%-RGO/TiO_2_/Ag and 1%-RGO/TiO_2_ photocatalysts, as well as the enlarged diagram of the peak split and combination where 2θ = 36–40.

Figure 4 shows the XRD patterns of RGO/TiO_2_ and RGO/TiO_2_/Ag powders. The RGO/TiO_2_/Ag powder shows characteristic peak of Ag_2_O at (111) crystalline face and characteristic peak of Ag at (111) crystalline face. This is mainly due to Ag nanoparticles is easily oxidized and produces metal oxides such as Ag_2_O^17^. Fortunately, the introduction of Ag_2_O in TiO_2_ will create p-n heterostructure, lower its band-gap width and enlarging the spectral response range of TiO_2_^33^. The introduction of Ag also helps lower the recombination rate of the photo-produced electron hole, thus improving the photocatalysis performance. In conclusion, the Ag/Ag_2_O combination in TiO_2_ can coordinate with each other and improve photocatalysis activity of TiO_2_.

### XPS and RAMAN analysis of photocatalysts

**Figure 5:**
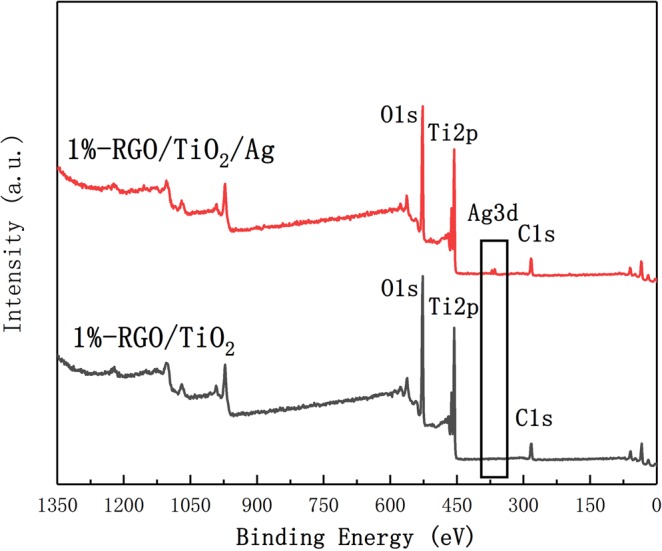
XPS total spectrogram of 1%-RGO/TiO_2_/Ag and 1%-RGO/TiO_2_ photocatalyst, with determination and remarking for major elements.

Figure 5 shows the XPS total spectrogram of 1%-RGO/TiO_2_/Ag and 1%-RGO/TiO_2_ powders. It can be seen from the figure that the characteristic peak of Ag (365 eV) shows in the 1%-RGO/TiO_2_/Ag powder, which means the Ag has been successfully introduced into the RGO/TiO_2_ system.

**Figure 6:**
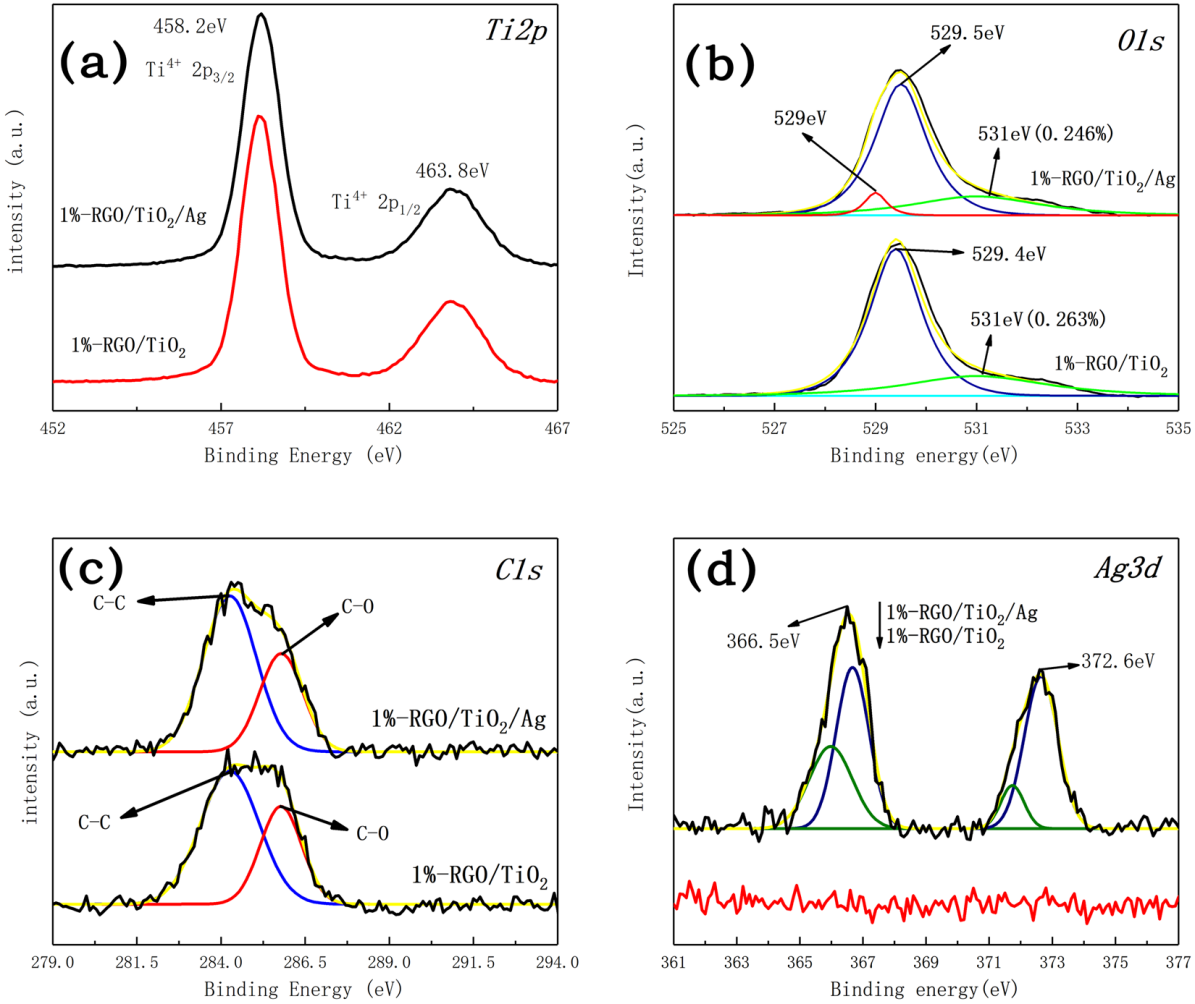
XPS comparison diagram for Ti2p elements of the two photocatalysts, with markings for electrons and peaks of th elements (**a**), XPS comparison diagram of peak split and combination for O1s elements of the two photocatalysts, with markings for the peak positions (**b**), XPS comparison diagram of peak split and combination for C1s elements of the two photocatalysts, with markings for bonding methods (**c**), XPS comparison diagram of peak split and combination for Ag3d elements of the two photocatalysts, with markings for the peak positions (**d**).

Figure 6(a) is an XPS chart of a titanium element of a photocatalyst doped with silver and undoped silver. Among them, titanium exists in the form of positive tetravalent. According to Fig. 6(b), the O element in 1%-RGO/TiO_2_ and 1%-RGO/TiO_2_/Ag powders is bonded as Ti-O-Ti and Ti-O-C, and the Ti-O-C bonding will create new energy level above TiO_2_ valence band, also lower the excitation wavelength of TiO_2_, thus improving the utilization ratio of TiO_2_^34^.

It can be seen from Fig. 6(c) that element C exists mainly in the form of C-C bonded C-O bonds, in which the C-C bond comes from the lamellar structure of graphene and the C-O comes from the bonding between graphene and titanium dioxide and the incompletely reduced oxygen-containing groups. Furthermore, the high-resolution XPS spectrum of Ag 3d for 1%RGO/TiO_2_/Ag samples is shown in Fig. 6(d). It can be seen from the graph that the silver element exists in two ways: Ag and Ag^+^. The calculation shows that the atomic number ratio of Ag/Ag^+^ is 1:1.1496.

**Figure 7:**
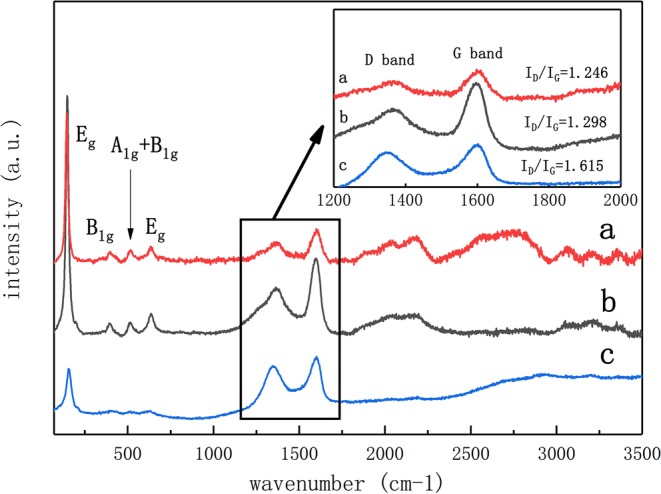
Raman comparison diagram for the three curves of (**a**–**c**) respectively represent the proportion of 1%-RGO/TiO_2_/Ag, 1%-RGO/TiO_2_ and 1%-RGO/TiO_2_ without high temperature treatment by tubular furnace.

Figure 7 is the Raman spectrum of 1%-RGO/TiO_2_/Ag and 1%-RGO/TiO_2_ powders and 1%-RGO/TiO_2_ gel without high temperature treatment. It shows the Eg, B1g, A1g + B1g and EG Raman characteristic peaks of anatase titanium dioxide at 147.2 cm^−1^, 397.2 cm^−1^, 515.2 cm^−1^ and 638.6 cm^−1^, where the characteristic peaks of anatase TiO_2_ are not obvious without thermal treatment, as the TiO_2_ is in long-term amorphous status^35^. After thermal treatment, the anatase Raman peak increases, and the amorphous TiO_2_ becomes anatase TiO_2_, which is the same as our previous work had shown^29^. The D-peak and G-peak of carbon atom crystal in graphene form at 1364.2 cm^−1^ and 1596.8 cm^−1^, which represent the lattice imperfection of the carbon atom and the in-plane stretching vibration of carbon atom sp^2^ hybridization. According to calculation, the I_D_/I_G_ value of RGO/TiO_2_ photocatalyst without thermal treatment (I_D_/I_G_ = 1.615) is far greater than that after thermal treatment (I_D_/I_G_ = 1.298), which means the restoration degree of the later is much better after thermal treatment^36^.

### TEM and HRTEM images of RGO/TiO_2_ and RGO/TiO_2_/Ag powders

**Figure 8:**
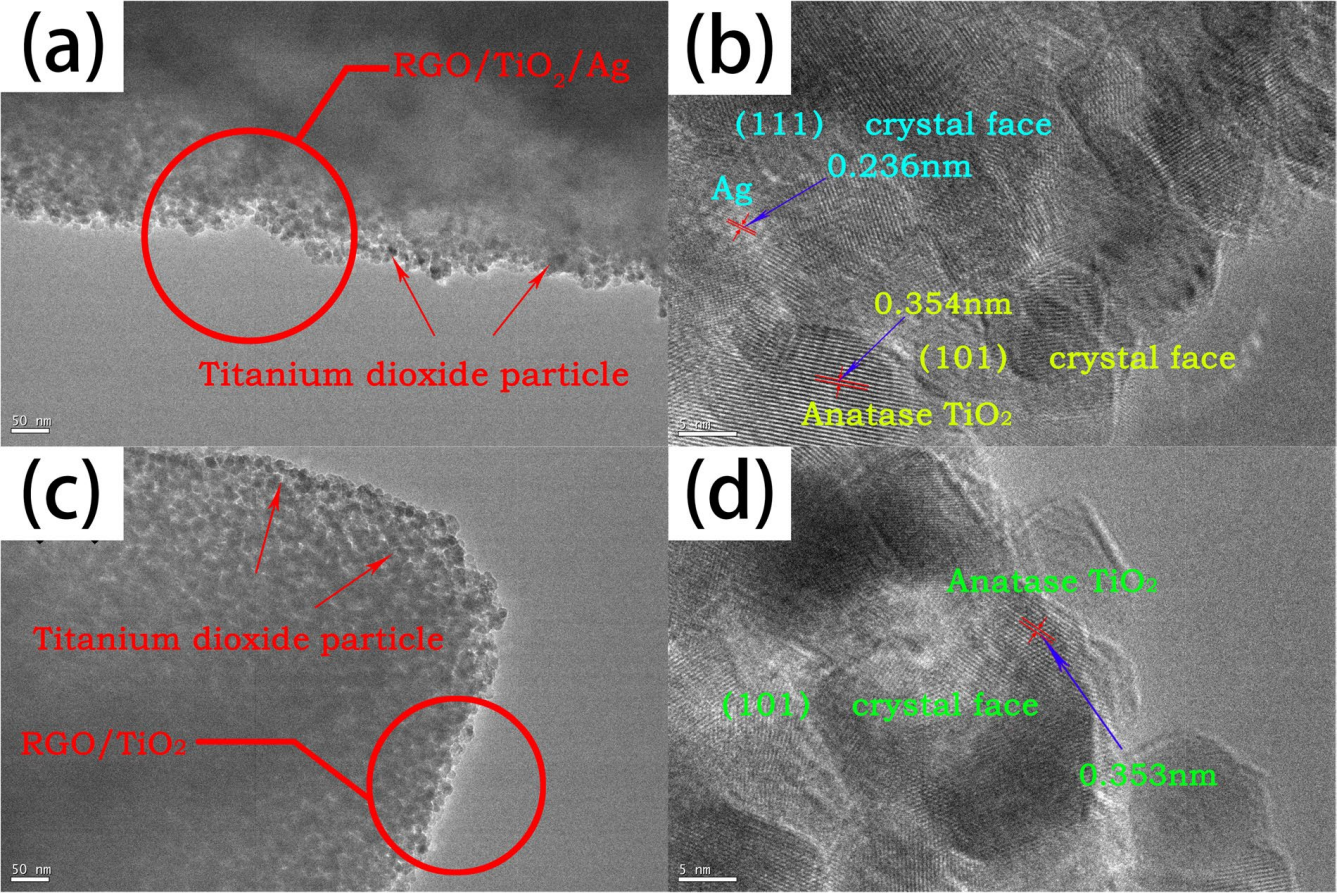
Morphology of 1%-RGO/TiO_2_ and 1%-RGO/TiO_2_/Ag catalyst powder. (**a**) Is the TEM photographs of 1% RGO/TiO_2_/Ag. (**b**) Is the HRTEM of 1% RGO/TiO_2_/Ag. (**c**) Is the TEM photographs of 1% RGO/TiO_2_. (**d**) Is the HRTEM of 1% RGO/TiO_2_.

Figure 8 is TEM and HRTEM images of 1%-RGO/TiO_2_ and 1%-RGO/TiO_2_/Ag powders. It can be seen from the Fig. 8(a–c) that titanium dioxide is uniformly loaded on the surface of RGO and its particle size is about 10nm-20nm. The interface between RGO and TiO_2_ is tightly bound as previously designed^29^, which is beneficial to the transmission of photo-generated electrons and improves the photocatalytic effect of the sample. In addition, crystalline faces of Ag (111) at a distance of 0.236 nm are also found in the 1%-RGO/TiO_2_/Ag system (Fig. 8(b)), which means Ag is deposited on the surface of titanium dioxide.

### The catalytic activity of photocatalyst

**Figure 9:**
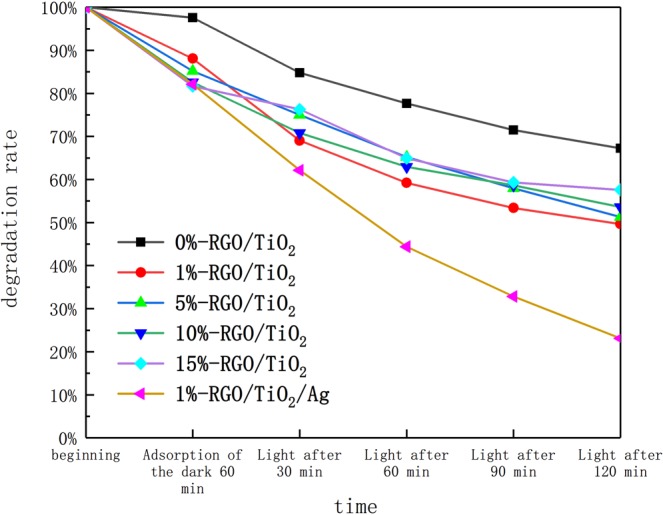
The absorbance of six kinds of photocatalysts under dark adsorption and ultraviolet illumination was measured after formaldehyde adsorption and catalytic degradation.

Figure 9 is a comparison of the degradation rates of liquid solutions catalyzed by different photocatalysts. In the dark adsorption stage, as the proportion of RGO increases, the concentration of formaldehyde in the liquid decreases because the π electrons enriched between the RGO layers contribute to the adsorption of formaldehyde molecules^37^. In the light phase, with the increase of the proportion of RGO, the degradation rate of formaldehyde by photocatalyst increased first and then decreased. When a small amount of graphene oxide is added to the titanium dioxide, the electron emission capability of the graphene oxide can promote the separation of photogenerated electron holes of the titanium oxide and improve the photocatalytic effect. Moreover, excessive addition of graphene oxide causes the light transmittance of the system to deteriorate, resulting in a decrease in degradation efficiency. Among them, 1%-RGO/TiO_2_ has the best effect on formaldehyde degradation.

When Ag is introduced, the degradation rate of formaldehyde is significantly increased. The photocatalytic reaction kinetics of TiO_2_ satisfies the Langmuir-Hinshelwood model^38,39^. The function equation of the model is shown in Eq. (2):

2 $$-\frac{{\rm{dC}}}{{\rm{dt}}}={\rm{k}}\frac{{\rm{K}}\cdot {\rm{C}}}{1+{\rm{K}}\cdot {\rm{C}}}$$

**Figure 10:**
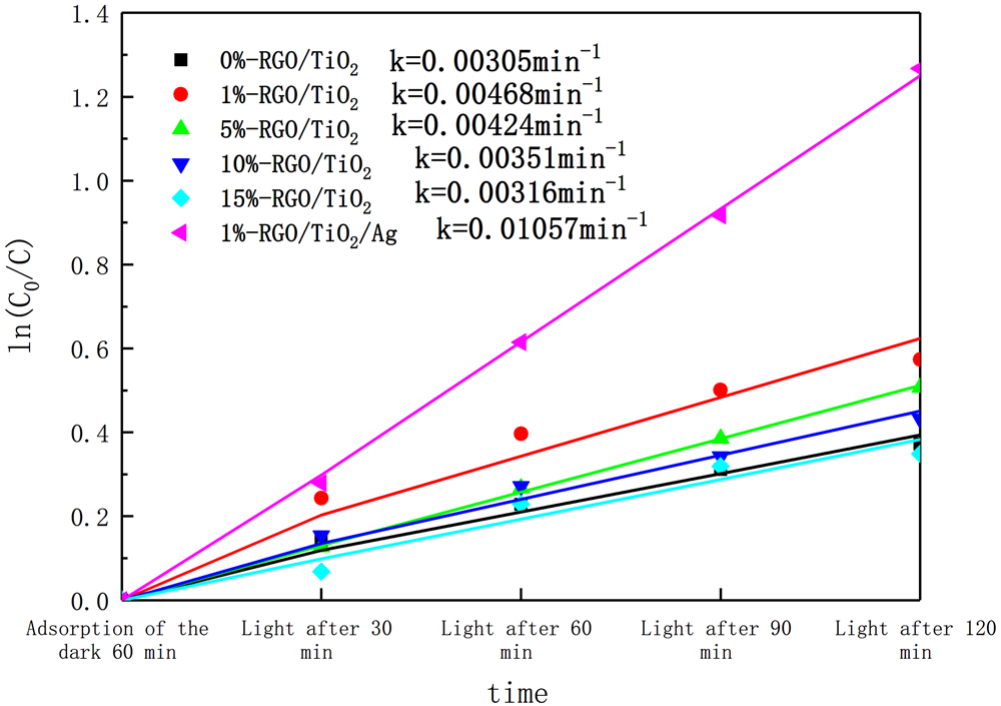
Six kinds of photocatalysts first-order reaction kinetics linear fitting curve.

When the concentration of the adsorbed molecules on the surface of the photocatalyst is less, K ∙ C ≪ 1, the above formula becomes −dC/dt = k ∙ K ∙ C, and the adsorption and degradation reaction of the photocatalyst on the adsorbed molecule satisfies the first-order reaction. According to Fig. 9, a first-order kinetic curve of the photocatalyst for degrading liquid formaldehyde of different components is plotted, as shown in Fig. 10. The concentration of each type of photocatalyst as a function of reaction time can well conform to the first-order kinetic equation and related laws. This is show that the degradation rate of formaldehyde by photocatalyst in the experiment is controlled by the diffusion of formaldehyde molecules. It was found that the photocatalyst after silver doping had the highest reaction kinetic constant and its value was 0.01057 min^−1^.

### BET surface areas and pore distributions of RGO/TiO_2_/Ag aerogel photocatalyst

**Figure 11:**
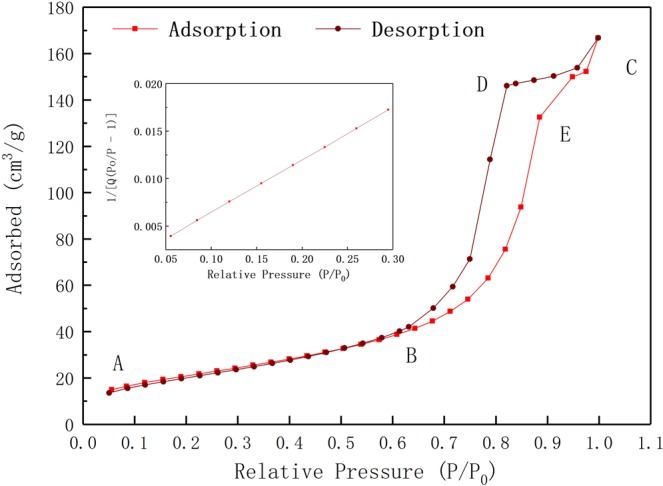
Absorption and desorption curve for 1%-RGO/TiO_2_/Ag aerogel photocatalyst in nitrogen atmosphere.

**Figure 12:**
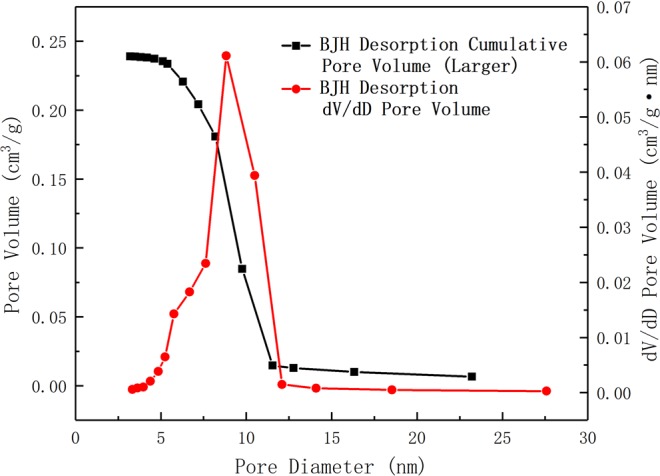
Pore size distribution of 1%-RGO/TiO_2_/Ag aerogel photocatalyst.

Figure 11 shows the absorption and desorption curves of RGO/TiO_2_/Ag aerogel, in which the absorption and desorption process are obviously lagged, with loop line at BC section. This means that there is mesopore in the aerogel. According to calculation, the specific surface area of the aerogel is 77.3672 m^2^/g. Figure 12 shows the aperture curves of the aerogel, in which the majority of aperture of the aerogel ranges from 7.6 to 12.1 nm, provide easy entry/exit for formaldehyde gas.

### SEM of RGO/TiO_2_/Ag aerogel photocatalyst

**Figure 13:**
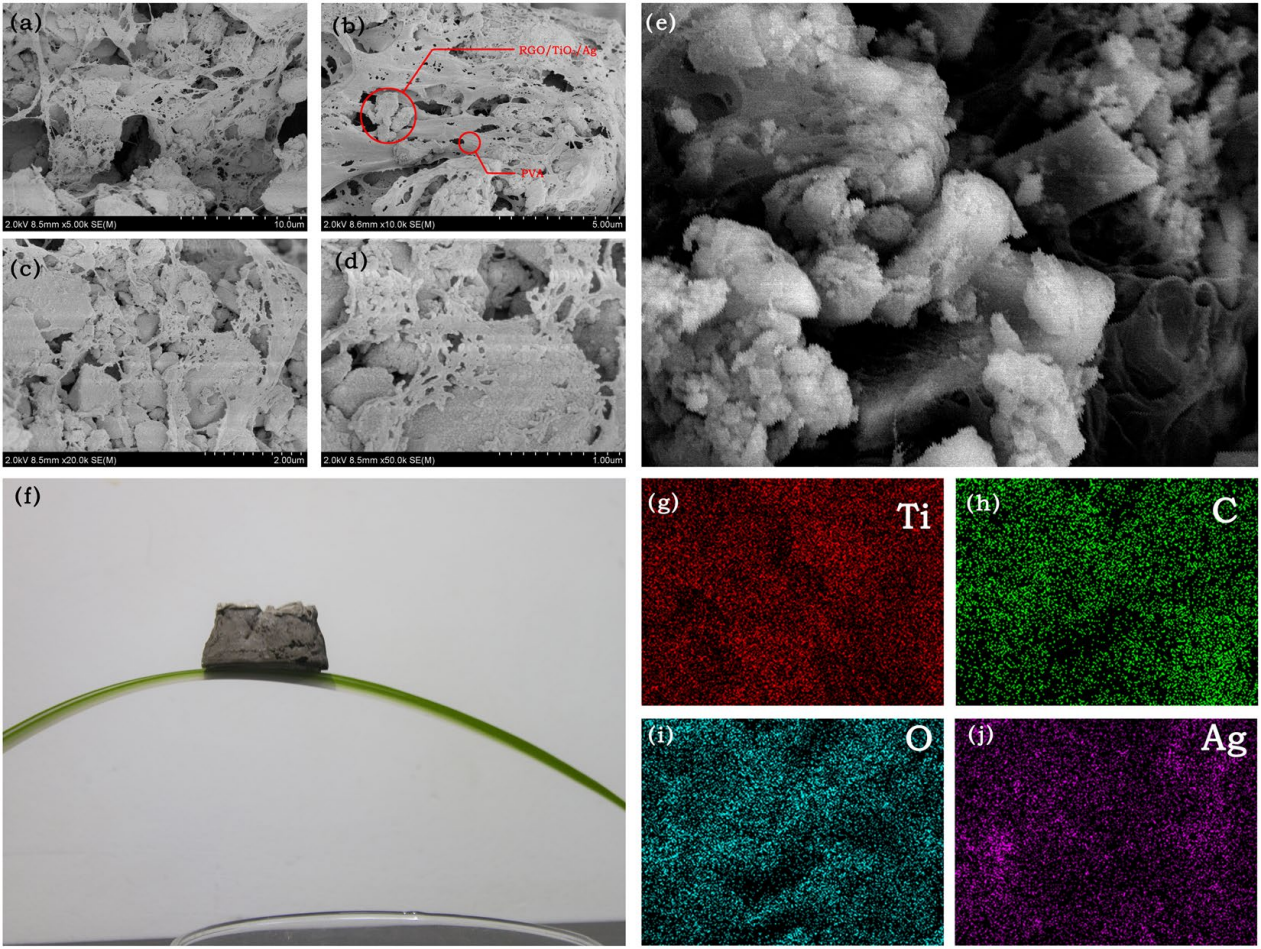
(**a**–**d**) SEM images of aerogel-type 1%-RGO/TiO_2_/Ag photocatalyst, (**e**) Electronic image of aerogel-type 1%-RGO/TiO_2_/Ag photocatalyst, (**f**) A static image of aerogel on plant leaves, and EDS elemental mappings of (**g**) Ti, (**h**) C, (**i**) O and (**j**) Ag elements of aerogel-type 1%-RGO/TiO_2_/Ag photocatalyst.

Figure 13(a) show the SEM images of 1%-RGO/TiO_2_/Ag aerogel photocatalyst, in which polyvinyl alcohol macromolecules entangles and bridges into a continuous and compact 3D webbing structure. In the meantime, Fig. 13(g) shows that the Ag spreading in the entire photocatalyst aerogel is quite even. This is because under photocatalytic conditions, silver ions combine with photogenerated electron reduction and grow *in situ* on the surface, which facilitates the transfer of electrons and the separation of electrons and holes in the photocatalytic process, improving its photocatalytic effect. It can also be seen from the actual photo of aerogel in Fig. 13(c) that the prepared aerogel floats on the blade, showing its characteristics of low density. This is resulted by the vast loosen mesoporous structure inside, making greater specific surface area and stronger absorption capability for the aerogel.

### Catalytic activity of aerogel photocatalyst

**Figure 14:**
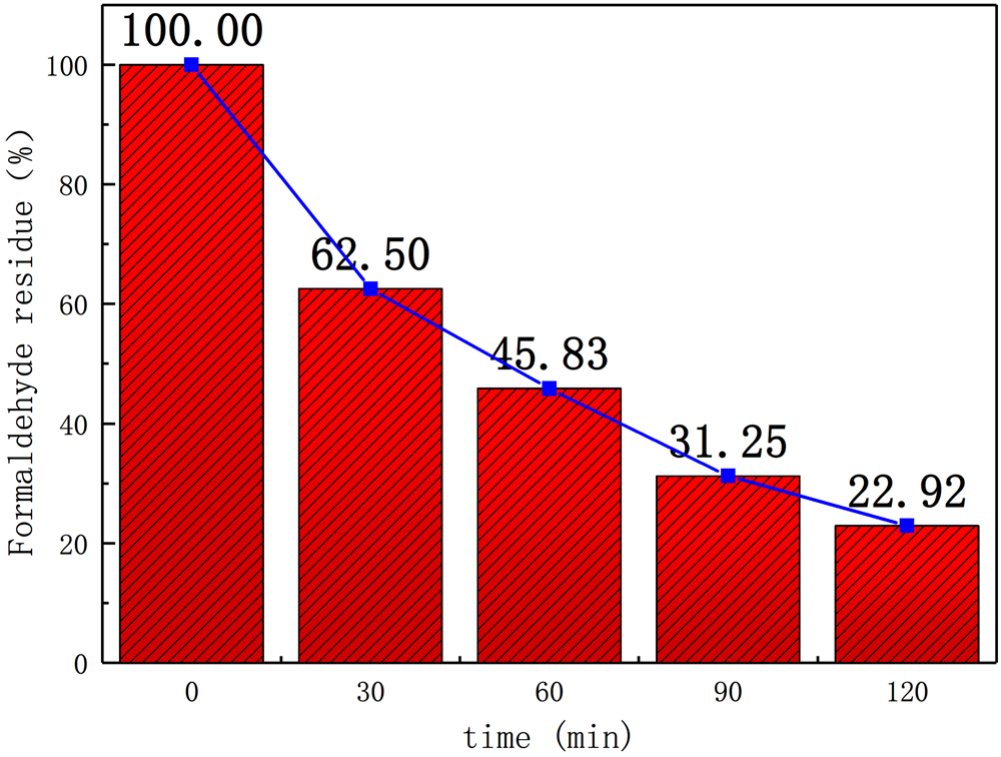
Diagram of formaldehyde residue rate after degradation by photocatalyst aerogel.

Figure 14 shows the diagram comparing the remaining percentage of formaldehyde gas against the duration of UV-lighting, after the degradation to the formaldehyde gas by the photocatalyst aerogel. According to the diagram, the degradation ratio of formaldehyde gas can reach 77.08% after being exposed under mercury lamp for 120 minutes. Based on the first-order kinetic Eq. (3):

3 $$\mathrm{ln}\,\frac{{C}_{0}}{C}={K}_{app}t$$

**Figure 15:**
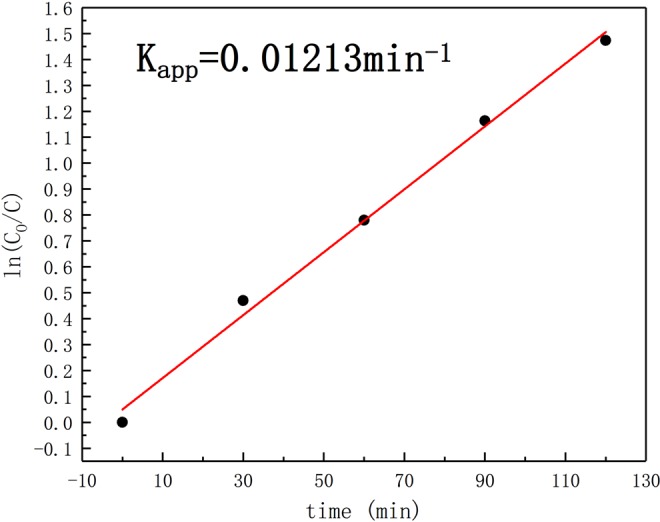
First-order kinetic curve of degradation for formaldehyde gas by photocatalyst areogel.

Figure 15 shows the first-order kinetic degradation curve for the photocatalyst aerogel, in which the degradation to formaldehyde gas is level 1 reaction, and the kinetic constant Kapp = 0.01213 min^−1^.

## Discussion

In order to solve the problems of low utilization rate of light energy and difficult recovery of TiO_2_ powder, the RGO/TiO_2_/(Ag) powders and RGO/TiO_2_/Ag aerogel photocatalyst were designed and prepared in this paper. The result show that anatase titanium dioxide in nano crystal structure can be created by applying thermal treatment at 400 °C, with the grain radius of TiO_2_ was 10 nm–20 nm. After introducing Ag into photocatalyst by UV-lighting irradiation method, XRD, XPS, TEM all prove that Ag/Ag_2_O system is successfully introduced into photocatalyst. After analyzing the catalysis performance of RGO/TiO_2_/Ag powder, it can be seen that the degradation speed is greatly improved after introducing Ag, which is because Ag can prevent photo-produced electron hole pairs from recombination. The creation of Ag_2_O also help lowering the bond-gap width of titanium dioxide.

The BET test shows that the aerogel has great specific surface area, with the mesoporous diagram ranging from 7.6 nm to 12.1 nm, which can be enough to breathe in/out the formaldehyde gas. According to SEM image, RGO/TiO_2_/Ag aerogel photocatalyst is evenly spread in the polyvinyl alcohol 3D webbing structure. The photocatalytic degradation of formaldehyde gas by RGO/TiO_2_/Ag aerogel showed that the degradation rate of formaldehyde was 77.08% after UV irradiation of 2 h.

## Material and Methods

### Characterization

Thermogravimetric analysis (Setsy Evolution) was used to analyze the sample powder. XRD patterns for obtained photocatalysts were recorded using a D8 advance X-ray diffractometer with Cu Kα radiation at a scanning rate of 5°/min. Particle size and morphology were determined using transmission electron nicroscopy (TEM, Hitachi S4800) and scanning electron microscope (SEM, TECNAI G2 (TF20)). The Brunauer-Emmett-Teller(BET) specific surface area of the samples were determined by using nitrogen adsorption with surface area analyzer (MicroActive for ASAP 2460). The elements in the samples were analyzed under radiation from Al target using the X-ray photoelectron spectroscopy (ESCALAB250). Raman signal (HORIBA LabRAM HR Evolution) was measured in the range of 50–4000 wavenumber using a laser source of 514 nm. The absorbance of the mixture of formaldehyde and acetylacetone was determined by visible spectrophotometer, and the model was sperm 722S. Aerogel photocatalyst is prepared by supercritical drying machine (FD-1A-50).

### Preparation of RGO/TiO_2_

GO was mixed with 9 g butyl titanate in 25 ml anhydrous ethanol. The content of graphene oxide in X%-RGO/TiO_2_ (X = 0,1,5,10,15) is 0 g, 0.002 g, 0.01 g, 0.02 g, 0.03 g respectively. Among them, X is the mass ratio of graphene oxide to titanium dioxide after conversion, and through constant temperature water bath stirring and ultrasonic treatment to mix it. Hydrochloric acid (1.8 ml), anhydrous ethanol (50 ml) and deionized water (12 ml) were stirred and mixed to obtain titrant. The mixture of GO and butyl titanate was titrated by the sol gel method until the mixture formed the gel. The gel aged after 6 h, and was dried under 70 °C, then was grinded into powder. After being treated by nitrogen atmosphere and heated in the tube furnace at 400 °C for 3 h, the RGO/TiO_2_ photocatalyst was generated.

### Preparation of RGO/TiO_2_/Ag photocatalyst

In an anhydrous ethanol solvent, preparation of 1%-RGO/TiO_2_ photocatalyst is made with silver nitrate in accordance with the quality ratio of 1 g: 0.03 g as the proportion of mixture. When treated with UV lamp (300 W) to reduce silver ions, the mixture is processed for centrifugation and drying operation. The solid powder is then heated in a tube furnace to dry in a nitrogen atmosphere. The RGO/TiO_2_/Ag photocatalyst is then produced.

### Preparation of RGO/TiO_2_/Ag aerogel

Then, in 0.3 g generated photocatalyst powder, 0.7 g polyvinyl alcohol solution was added (mass fraction 1.5%) as PVA solution. The mixture was kept in refrigerator under −20 °C. The solution was continuously stirred until frozen into a mass. In the supercritical drying machine after 30 h of freeze drying under −40 °C, finally the RGO/TiO_2_/Ag aerogel is made.

### Degradation of liquid formaldehyde

The photocatalytic activity of RGO/TiO_2_ photocatalyst and RGO/TiO_2_/Ag photocatalyst was evaluated by analyzing the removing of formaldehyde solution at room temperature. During this process, the mass volume ratio of photocatalyst powder and formaldehyde solution was 0.5 g: 30 ml, and the formaldehyde solution was 32 mg/L. The mixture was loaded into the quartz tube. The mixture of formaldehyde and photocatalyst was placed in the dark for 1 h to reach adsorption/desorption balance, and then was stirred continuously under ultraviolet light. Exposed under a 300 W mercury lamp, 2.5 ml of formaldehyde solution was filtered out every 30 minutes, and diluted for 10 times, then added 2.5 ml of acetylacetone solution. The residual concentration of formaldehyde molecule was determined by detecting the change of absorbance at 414 nm. Acetylacetone chromogenic solution was prepared by mixing three kinds of samples: ammonium acetate (125 g), glacial acetic acid (15 ml) and acetone (1.25 ml), and then adding deionized water to 250 ml.

### Degradation of gas formaldehyde

The photocatalytic activity of RGO/TiO_2_/Ag aerogel photocatalyst at room temperature was reflected by the change of formaldehyde concentration. The degradation process was realized by UV- lighting to the photocatalyst in the 2L total closure device contains 3ppm formaldehyde gas. The internal gas was collected every 30 min and the obtained gas was dissolved in water to produce the corresponding concentration of formaldehyde liquid. Determination of the concentration of formaldehyde liquid by acetylacetone method. Based on equation (4):

4 $${\rm{\eta }}=(1-{C}_{t}/{C}_{0})\ast 100 \% $$

Can calculate the degradation rate of gaseous formaldehyde, where η is the degradation rate of gaseous formaldehyde

## Data Availability

No datasets were generated or analyzed during the current study.
